# An Embryonic Field of Study: The Aquatic Fate and Toxicity of Diluted Bitumen

**DOI:** 10.1007/s00128-017-2239-7

**Published:** 2017-12-14

**Authors:** Ftoon Alsaadi, Peter V. Hodson, Valerie S. Langlois

**Affiliations:** 1 Biology Department, Queen’s University, Kingston, ON Canada; 2 School of Environmental Studies, Queen’s University, Kingston, ON Canada; 3 Chemistry and Chemical Engineering Department, Royal Military College of Canada, Kingston, ON Canada; 4 Institut National de la Recherche Scientifique - Centre Eau Terre Environnement (INRS-ETE), 490 de la Couronne, Quebec City, QC G1K 9A9 Canada

**Keywords:** Ecotoxicology, Pipeline, Polycyclic aromatic hydrocarbon, Diluted bitumen (dilbit), Fish

## Abstract

Canada has experienced a significant increase in the transport of diluted bitumen (dilbit), a predominant oil sands product that combines bitumen with diluents derived from oil–gas condensates and other proprietary compounds. The proportion of diluent and the chemical composition of dilbit vary to meet seasonal transport requirements. While the toxic effects of a variety of crude and refined oils are well-studied, the toxicity of dilbit to aquatic species is less well known. This focused review summarizes dilbit production, chemistry, and the few data on toxicity to aquatic species. These data suggest that un-weathered dilbit would cause effects on fish equivalent to those of conventional oils, but its toxicity may be lower, depending on interactions among test conditions, the behavior of dilbit added to water and the species tested.

## Introduction

Bitumen is a highly-weathered oil extracted from natural oil sands deposits, primarily in Canada, Venezuela, Kazakhstan, and Russia (Meyer et al. 2007). In Canada, oil sands extend from Saskatchewan west to British Columbia, with most extraction occurring in Alberta. Following extraction, some bitumen may be shipped by rail as is (neatbit), or by pipeline or rail as bitumen diluted with synthetic crude produced by hydrocracking (synbit) (Dew et al. 2015). However, most is shipped by pipeline as diluted bitumen (dilbit) after adding diluent to facilitate flow. Although Dew et al. (2015) provided a comprehensive review of the production and transportation of bitumen products, no studies of the toxicity of neatbit or synbit were found, so this review concerns the aquatic fate and effects of spilled dilbit.

Dilbit, like crude oil, is composed primarily of hydrocarbons, including saturates, aromatics, resins and asphaltenes (King et al. 2017). Unlike crude oil, dilbit is also made of low molecular weight (LMW) saturates and mono- and di-aromatics that are derived mainly from oil–gas condensates, which are proprietary diluents; complete lists of ingredients are not published. Aromatics include polycyclic aromatic compounds (PACs), which are non-polar, hydrophobic and associated with the chronic toxicity of oil to fish embryos (e.g., polycyclic aromatic hydrocarbons (PAHs); Adams et al. 2014). PACs also include heterocyclic aromatic compounds, such as dibenzothiophenes, for which there are few toxicity data. PAHs that contain alkyl substituents (alkyl-PAHs) predominate in oil and are embryotoxic (Hodson 2017). In this review, the term ‘PAH’ is used frequently because most publications report oil toxicity in terms of the summed concentrations of all PAHs measured, or total PAHs (TPAHs).

Oil transportation creates a risk of accidental spills, and fish species inhabiting rivers and lakes along current and proposed pipeline and rail routes are vulnerable to oil exposure and toxicity (Dupuis and Ucán-Marín 2015). Thus, it is important to understand the potential consequences of dilbit spills to freshwater environments. Although the toxicity of conventional oils to aquatic species is well established, few studies have assessed the effects of dilbit blends. This focused review provides an overview of (1) Canada’s oil industry, (2) the production of dilbit, (3) the physical and chemical components of dilbit, (4) the effects of PACs on aquatic species, and (5) detoxification and PAH metabolism.

*Canada’s current oil industry* Canada has the world’s third largest oil reserves, after Venezuela and Saudi Arabia (Olsson 2012) and is currently the fifth largest producer and fourth largest exporter of crude oil. In 2013, Canada exported 74% of its production, most to the United States (NRCan 2014). Canadian production includes conventional crude and heavy oils, bitumen (as dilbit), and condensate. Of these, oil sands bitumen represents 97% of the petroleum produced (CAPP 2016). Among Canadian provinces, Alberta generates nearly 80% of Canada’s oil and is the world’s largest producer of oil sands bitumen.

*Production and transportation of bitumen from oil sand* Oil sands bitumen is a semi-solid or solid, and is recovered by mining or in situ steam extraction (Read and Whiteoak 2003). For deposits less than 75 m below the surface, surface mines send bitumen to processing plants, where it is mixed with hot water and separated from sand by gravity. Deeper deposits are extracted in situ by injecting steam to reduce bitumen viscosity, enabling pumping to the surface (Grant and Myers 2004). To facilitate transportation, bitumen is diluted approximately 30% with natural gas condensates or other light hydrocarbons, resulting in a range of dilbit blends, depending on seasonal requirements for viscosity (Dupuis and Ucán-Marín 2015).

*Pipeline transportation of oil and inland oil spills* In Canada, petroleum products are transported primarily via pipelines, the most efficient way to connect large supply basins to markets (NRCan 2014). Currently, more than 840,000 km of oil pipelines in Canada move approximately 1.3 billion barrels per year, valued at more than $100 billion (NRCan 2016). Most pipelines connect extraction sites in Alberta to refineries (NAS 2015), and larger inter-provincial and international pipelines deliver oil to markets (CEPA 2014). Canada’s current exports of dilbit to the United States exceed pipeline capacity, requiring some shipment by rail, and there are plans to add new pipelines, such as the Keystone XL pipeline to Cushing, Oklahoma (ECCC 2013; King et al. 2014). While pipelines are a relatively inexpensive and safe means of transportation, adding new pipelines increases the potential for terrestrial and freshwater spills because the quantity of oil shipped will also increase. According to the Transportation Safety Board of Canada (2016), 28% of 67 oil spills between 2007 and 2016 were from pipelines.

Because pipelines traverse watersheds for wetlands, streams, and lakes, spilled oil can enter water directly, or indirectly via groundwater transport or overland flow (NAS 2015). Once oil enters water, it is more difficult to recover because it can move quickly away from the release site. The precise effects of an oil spill will depend on the chemical and physical properties of the oil, the volume spilled, weather conditions, and the spill location (Chang et al. 2014). Apart from a 2007 spill of diluted synthetic crude to Burrard inlet (Dew et al. 2015), the first significant dilbit spill occurred July 26th, 2010, when an Enbridge pipeline burst and spilled approximately 4.24 million litres into Talmadge Creek, a tributary of the Kalamazoo River near Marshall, Michigan (USEPA 2013). The spill contaminated an estimated 40 km of the Kalamazoo River. The diluent volatilized quickly and up to 20% of the residual bitumen adhered to suspended sediments, thereby contaminating vegetation, bottom sediments, and shorelines as the sediments settled onto solid substrates (Murphy 2012; USEPA 2013). As of mid-2013, roughly 684,000 litres remained submerged in the river (USEPA 2013).

*Physical properties of bitumen* Important physical properties of bitumen include viscosity and density, which are determined by the proportion and types of large polar molecules, such as resins and asphaltenes (NAS 2015). A greater percentage of lighter components, such as saturates, results in lower viscosities and densities (King et al. 2017). The density of bitumen exceeds 1.0 g/mL (Lee et al. 2015), and it will sink in fresh water, whereas the density of dilbit ranges from 0.92 to 0.93 g/mL and it floats in fresh water. However, dilbit may sink after weathering when light components evaporate, as occurred in the Kalamazoo River (Lee et al. 2015).

*Chemical properties of bitumen* Oil is a complex mixture of hydrocarbons, including saturates, aromatics, resins, and asphaltenes, as well as metals and compounds containing nitrogen, sulphur, and oxygen (Dupuis and Ucán-Marín 2015). Saturates are usually the most common components, the most biodegradable, and the least toxic (Fan and Buckley 2002; Adams et al. 2014). Aromatics are non-polar and hydrophobic, and include monoaromatics (benzene, toluene, ethylbenzene, and xylenes; BTEX) and PACs with two or more rings. PACs are relatively insoluble in water, adsorb to particles, and settle to aquatic sediments. Resins contribute to the adhesive properties of oil; they are polar and may include sulphur, nitrogen, and oxygen (Speight 2002). Finally, asphaltenes are high molecular weight (HMW; > C30) compounds that contribute to viscosity and density in heavy oils and bitumen (Akmaz et al. 2011).

Compared to conventional crude oil, dilbit contains more carbon than hydrogen, lower amounts of LMW compounds, and higher amounts of resins and asphaltenes (Lee et al. 2015). Thus, it is more adhesive and generates more persistent residues that adhere to shorelines and structures (NAS 2015). Dilbit also has a lower total PAH content, but a higher proportion of three- to five-ringed alkylated PAHs (e.g., King et al. 2017). The most commonly transported dilbit products in Canada (by volume) are Access Western Blend (AWB) and Cold Lake Blend (CLB; King et al. 2014). AWB contains a higher proportion of HMW compounds than CLB, though the latter has a higher proportion of saturates and PACs (King et al. 2014), and thus may be more toxic to fish.

*Fate and behaviour of dilbits in water* The fate and behaviour of spilled oil are affected by a sequence of physical and chemical changes called weathering (Wang and Fingas 2003), which begin immediately after a spill and can proceed rapidly (NAS 2015). Physical processes include spreading, dispersion, emulsification, adhesion, and sedimentation, while chemical processes include photo- and biodegradation. Physical–chemical processes, which do not alter molecular structures, include evaporation and dissolution of LMW compounds (alkanes, and mono- and di-aromatics < C10), adsorption to particulates and dissolved organic matter, and bioaccumulation by aquatic organisms. Fate and behaviour can also be affected by environmental factors, such as salinity, temperature, and turbidity, and the extent of weathering varies with the density, viscosity, chemical composition, and adhesiveness of the fresh oil (Lee et al. 2015).

The fate and behaviour of spilled dilbits differ from conventional crude oils due to their unique physical and chemical properties (Tsaprailis 2013), and weathering enhances these differences. As with conventional crude oils, un-weathered dilbits have a lower density than water and will float (NAS 2015), potentially contaminating shorelines and aquatic birds and mammals. Although this behaviour was evident following the Kalamazoo River spill (Lee et al. 2015), the rapid evaporation of diluent increased the density of dilbit and facilitated its entrainment into the water column as droplets, which subsequently adsorbed to particulates and contaminated bottom sediments. In contrast, weathered heavy oil approaches the density of fresh water, but is less likely to sink than dilbit (NAS 2015). The rapid loss of diluent also creates a fire hazard. The temperature at which dilbit loses sufficient vapours to become flammable is termed its ‘flash point’, which is lower than that of conventional oils and bitumen (Fingas 2015). However, the residual dilbit is less flammable and more viscous than fresh dilbit or heavy oil (NAS 2015).

Oil weathering, fate, and behaviour are also affected by water temperature and turbidity. Oil that flows well at higher temperatures is more viscous and slow-moving at lower temperatures, which reduces the weathering rate. Conversely, wave action increases the rate at which oil droplets are entrained into the water column and the partitioning of soluble hydrocarbons from oil to water (NRCan 2014), and increases the density of residual oil droplets. In calm water, prolonged exposure to air enhances diluent evaporation and increases the viscosity and density of residual oil to that of the parent bitumen (Lee et al. 2015).

Because dilbits contain a similar array of hydrocarbons as conventional heavy crude oils, the toxicity of dilbit should be similar. However, even toxicity tests may be affected by the rapid evaporation of oil–gas condensates. After prolonged stirring of dilbit-water mixtures to prepare water accommodated fractions (WAFs), the loss of volatiles and associated changes in viscosity and dispersibility appear to reduce the concentrations and measured toxicity of PACs in test solutions (Madison et al. 2017). As a consequence, dilbit may appear less toxic to aquatic species than crude oils, largely due to lower exposure to the toxic components of dilbit, rather than to a lower toxicity of the components.

*Toxicity of dilbit* The constituents of oil commonly associated with oil toxicity to fish are LMW aliphatics and aromatics (< C10; most water soluble and volatile) and larger three- to six-ringed PAHs (C10-C30); aliphatics > C10, resins and asphaltenes have not been associated with toxicity (Adams et al. 2014). LMW compounds, including naphthalene (two-ringed PAH), typically cause only acute narcosis (lethality within 24 h), primarily because concentrations in test solutions can decline rapidly with weathering (Redman and Parkerton 2015). However, there are few studies of the delayed effects of brief exposures to un-weathered oils, and no studies of toxicity following chronic exposures to oil–gas condensates used as diluents (Lee et al. 2015).

The chronic embryotoxicity of PAHs with three or more rings increases with hydrophobicity, as expressed by the octanol–water partition coefficients (K_ow_; Hodson 2017). Hydrophobicity increases with molecular size and determines the rate of water–lipid partitioning of PAHs, their accumulation by fish, and their apparent toxicity. However, acute and chronic toxicity of large PAHs (> 5 rings) may not increase at log Kow values > 6. For industrial organic compounds, low water solubility and large molecular size limit the rates of water-membrane partitioning and the dose accumulated (toxicity cut-off; Veith et al. 1983).

The interaction of PAHs with specific cellular receptors also varies with the number, size, and distribution of alkyl side chains (Billiard et al. 2002). Thus, variations of chronic toxicity among PAHs reflect differences in both exposure and molecular interactions within cells. Heterocyclic aromatics are also embryotoxic (e.g., dibenzothiophene; Rhodes et al. 2015), but there are too few comparative studies to determine how sulfur, oxygen, or nitrogen in aromatic rings change toxicity. Most publications express the embryotoxicity of oil as TPAHs in test solutions. However, the TPAH values reported for median lethal (LC50) and median effective (EC50) concentrations may not reflect the concentrations of those PAHs that are actually toxic. For example, naphthalenes are included in TPAHs and are acutely lethal, but are not associated with chronic toxicity (Adams et al. 2014); chrysenes are chronically toxic but contribute little to acute lethality (Lin et al. 2015). The list of PAHs analyzed varies among studies, which can bias estimated toxicity. There is a need to measure a consistent list of PAHs in test solutions for acute and chronic exposures to allow more accurate comparisons of toxicity among studies.

WAFs of weathered Western Canadian Select (WCS) and CLB dilbit were not acutely lethal to fathead minnows and Atlantic silversides, but the 96 h LC50s for un-weathered dilbits ranged from 5.9 to > 16.3 mg/L of total petroleum hydrocarbons (TPH) (Barron et al. 2018); 96 h LC20s ranged from 4.0 to 15.7 mg/L TPH. WCS appeared somewhat more toxic than CLB, and marine silversides appeared more sensitive to dilbit than freshwater fathead minnows, as did freshwater (*Ceriodaphnia dubia*) and marine (*Americamysis bahia*) invertebrates. Lethality correlated well with TPH concentrations (LC50s = 4–16 mg/L), but less so with TPAH (8–40 µg/L) or BTEX (0.7–16 mg/L), and fell within the range of reported toxicities of conventional crude oils (Barron et al. 2018). In comparison to two conventional crude oils, dilbit was less toxic to zebrafish embryos when percent mortality over 7 days was compared to total concentrations of BTEX (un-specified dilbit blend; Philibert et al. 2016). The opposite was true when mortality of zebrafish embryos exposed to dilbit was compared to mortality of embryos exposed to TPAH concentrations. Rates of mortality increased from background between 10 and 18 mg/L BTEX and 20–50 µg/L TPAH. Medaka embryos may be less sensitive than other species; exposure to CLB and AWB did not cause mortality at TPAH concentrations up to 63 μg/L (Madison et al. 2015, 2017).

Bitumen sampled from stream sediments in the oil sands region of Alberta caused sub-lethal toxicity to fathead minnow and common white sucker embryos that closely resembled blue sac disease (BSD: craniofacial malformations; pericardial and yolk sac edema; spinal curvatures; fin erosion; haemorrhaging; induction of cytochrome P450 enzymes) (Colavecchia et al. 2004, 2006). BSD was observed in embryos exposed to crude oil or to individual PAHs (Ramachandran et al. 2004; Hodson 2017), but the frequency and severity of each sign varied among species (Lee et al. 2015). The phenotypic effects of oil exposure have been attributed to cardiotoxicity (Brette et al. 2014) linked to the blockage of potassium and calcium channels and leading to the disruption of normal heart conduction (Incardona 2017). This malfunction precedes other deformities, such as pericardial and yolk sac edemas (Incardona et al. 2014). Other effects of oil exposure may include neurological and behavioural defects (Philibert et al. 2016). At a cellular level, BSD includes dysregulation of a number of cellular pathways, including detoxification.

Dilbit exposure also causes BSD, with craniofacial malformations and yolk sac and pericardial edemas the most common signs observed in medaka (Madison et al. 2015, 2017), and pericardial edema the primary response of zebrafish (Philibert et al. 2016). Sockeye salmon parr exposed to CLB dilbit (summer blend) for 1 and 4 weeks exhibited concentration–dependent (3.5–66.7 µg/L TPAH) cardiac remodeling that correlated with reductions in swimming performance that could ultimately affect migratory success (Alderman et al. 2017a). Exposed salmon may also be less capable of exercise recovery than controls. Dilbit-treated and exercised fish experienced cell damage, as indicated by increased protein leakage from tissues, and inflammatory responses (Alderman et al. 2017b). These effects corresponded to signs of oxidative stress in trout embryos exposed to retene (Bauder et al. 2005), a C-4 phenanthrene found in crude oil.

Impairment of swim bladder inflation was also observed in over 50% of malformed medaka treated with dilbit, suggesting toxic effects on the autonomous neural control of inflation, or impaired access to the water’s surface by surface oil films (Madison et al. 2017). The latter mechanism was suggested by a failure of swim bladder inflation in control fish exposed to Nujol, a non-toxic mineral oil. However, other chemicals also impair inflation, perhaps acting through AhR-PAH inhibitory crosstalk with the Wnt signaling pathway, cascading to swim bladder malfunction (Jönsson et al. 2012). Other effects of dilbit included slight delays in the hatching of zebrafish, and decreased shelter-seeking behaviour at TPAH concentrations of 46 μg/L (Philibert et al. 2016). Both responses were caused by conventional crude oils, but dilbit appeared less toxic.

To date, most studies of dilbit toxicity have addressed effects on fish embryos. Given that sensitivity to oil toxicity varies considerably among early life stages, including gametes, embryos, and free-swimming embryos (McIntosh et al. 2010), further studies should assess the full life cycle of fish. Although embryos may survive chronic toxicity tests, BSD is associated with impaired development and growth, increased susceptibility to predation, and reduced survival to adulthood (Carls and Thedinga 2010). For example, the spawning runs of pink salmon that survived oil exposure as embryos were reduced by 40% compared to controls (Heintz et al. 2000). These findings highlight the ecological importance of the timing of dilbit spills, a major factor determining the impact on different life stages of each fish species.

*Detoxification and PAH metabolism* The molecular shape, the number, size, and location of alkyl substituents, and K_ow_ influence the aryl hydrocarbon receptor (AhR) binding affinity. For some PAHs (e.g., phenanthrenes), alkylated congeners are more potent CYP1A inducers because they bind more readily to the AhR than unsubstituted PAHs (Billiard et al. 2002). Significant and similar 15-fold increases of *cyp1a* mRNA levels were measured in medaka embryos exposed to either AWB or CLB dilbit (Madison et al. 2015, 2017), suggesting AhR activation by PAHs in dilbit blends. The expression of *cyp1a* was strongly correlated to the prevalence of BSD in medaka embryos (*R* ^2^ = 0.91; Madison et al. 2017), demonstrating that induction of *cyp1a* transcripts is a sensitive biomarker of oil exposure and PAH accumulation, and predictive of embryotoxicity (Carls et al. 2005). Expression of *cyp1a* in whole medaka embryos exposed to dilbit, responded to waterborne TPAH concentrations as low as 0.4 μg/L (Madison et al. 2017). Likewise, hepatic CYP1A enzyme activity was increased 15-fold in sockeye salmon exposed to CLB at concentrations ranging from 3.5 to 67 μg/L TPAH, and *cyp1a* expression in heart muscle increased significantly at 16.4 μg/L TPAH or higher (Alderman et al. 2017a). The oxygenation of PAHs by CYP1A enzymes can significantly change the toxicity of PAHs, which represents the balance between accelerated excretion of PAHs (detoxification) and the formation of toxic metabolites (activation; Hodson 2017).

## Conclusions

 In laboratory tests, exposure of early life stages of fish to un-weathered dilbit causes similar molecular, physiological, and phenotypic signs of toxicity as exposure to conventional crude oils. Dilbit toxicity appears lower than that of conventional crude oil, but comparisons are limited by differences in species tested and the effect of dilbit weathering during solution preparation on PAC concentrations in test solutions. The higher proportions of resins and asphaltenes in bitumen compared to conventional crude oils may also affect the partitioning of hydrocarbons from dilbit to water and the overall exposure of fish. Given the increasing transport of dilbit and related products by pipelines and rail, it is imperative to further investigate their potential effects on aquatic species and to understand the interactions between their chemical and physical properties and the extent to which all life stages of fish are exposed to PACs.
